# Optimization of Storage Temperature for Retention of Undifferentiated Cell Character of Cultured Human Epidermal Cell Sheets

**DOI:** 10.1038/s41598-017-08586-7

**Published:** 2017-08-15

**Authors:** Catherine J. Jackson, Sjur Reppe, Jon R. Eidet, Lars Eide, Kim A. Tønseth, Linda H. Bergersen, Darlene A. Dartt, May Griffith, Tor P. Utheim

**Affiliations:** 1 Department of Medical Biochemistry, Oslo University Hospital, Oslo, Norway; 2 Institute of Oral Biology, Faculty of Dentistry, University of Oslo, Oslo, Norway; 3 Department of Plastic and Reconstructive Surgery, Oslo University Hospital, Oslo, Norway; 4 Department of Ophthalmology, Oslo University Hospital, Oslo, Norway; 5 Institute of Clinical Medicine, Faculty of Medicine, University of Oslo, Oslo, Norway; 6 Schepens Eye Research Institute, Massachusetts Eye and Ear, Department of Ophthalmology, Harvard Medical School, Boston, USA; 7 Maissonneuve-Rosemont Hospital Research Centre and Dept. of Ophthalmology, University of Montreal, Montreal, Canada

## Abstract

Cultured epidermal cell sheets (CES) containing undifferentiated cells are useful for treating skin burns and have potential for regenerative treatment of other types of epithelial injuries. The undifferentiated phenotype is therefore important for success in both applications. This study aimed to optimize a method for one-week storage of CES for their widespread distribution and use in regenerative medicine. The effect of storage temperatures 4 °C, 8 °C, 12 °C, 16 °C, and 24 °C on CES was evaluated. Analyses included assessment of viability, mitochondrial reactive oxygen species (ROS), membrane damage, mitochondrial DNA (mtDNA) integrity, morphology, phenotype and cytokine secretion into storage buffer. Lowest cell viability was seen at 4 °C. Compared to non-stored cells, ABCG2 expression increased between temperatures 8–16 °C. At 24 °C, reduced ABCG2 expression coincided with increased mitochondrial ROS, as well as increased differentiation, cell death and mtDNA damage. P63, C/EBPδ, CK10 and involucrin fluorescence combined with morphology observations supported retention of undifferentiated cell phenotype at 12 °C, transition to differentiation at 16 °C, and increased differentiation at 24 °C. Several cytokines relevant to healing were upregulated during storage. Importantly, cells stored at 12 °C showed similar viability and undifferentiated phenotype as the non-stored control suggesting that this temperature may be ideal for storage of CES.

## Introduction

Since the first treatment of massive area burns in 1984^1^, use of cultured epidermal sheets (CES) for patients with burns has become routine in many burn treatment units^2^. CESs are used as both allogenic and autologous transplants. Undifferentiated cells within CES have been shown to respond to new signals from the local environment following transplantation^3^. They have been used to restore a clear corneal epithelium in a goat model of wounded cornea (limbal stem cell deficiency)^4^ and to reconstruct urethral epithelium in a rabbit model of urethral injury^5^. Adult epidermal stem cells have been shown to be capable of differentiating to all three germ layers when inserted into a mouse blastocyst^3^. Skin is therefore an attractive alternative source of autologous stem cells for regenerative medicine applications as it is highly abundant and easily accessible^6^.

Whether for use in treatment of skin burns or regeneration of other epithelia, expanded cells require appropriate storage conditions to maintain viability and phenotype for clinical application. Short-term storage can expand the utility of CES by providing flexibility in timing of transplant operations, back-up sheets for repeat operations, wider distribution, and an extended window for quality control and sterility testing in centralized culture facilities^7^. Storage needs are currently met by cryopreservation, which entails a complicated freeze/thaw schedule. Studies have also shown that the quality of cryopreserved CES upon thawing is variable^8, 9^. Here, we seek to extend the availability and use of CES for application in regenerative medicine by developing a short-term xenobiotic-free storage system that maintains CES quality and is convenient to use.

Retention of undifferentiated cell phenotype in cultured and stored CES is important for the treatment of patients with burns^10^. Likewise, transplantation of a high percentage of progenitor cells within transplanted cultured limbal epithelial cell sheets in the treatment of limbal stem cell deficiency results in a higher rate of clinical success^11^. Highly proliferative cycling epidermal progenitor cells are the first to contribute to regeneration following transplantation, while quiescent SCs provide long-term renewal^12^. Our objective was therefore to maintain an undifferentiated cell phenotype and proliferative capacity within CES during storage.

We have previously shown that temperature has a significant impact on the quality of stored cultured cells from a variety of tissues^13–16^. Based on analyses of phenotype (best at 12 °C) and viability (best at 24 °C) of CES in our two-week storage study^17, 18^, we hypothesized that 12 °C may be most promising for retention of proliferative capacity and undifferentiated cell phenotype in CES following one-week of storage. Therefore, in-depth analyses were carried out herein to compare one-week storage of CES stored at temperatures 4 °C, 8 °C, 12 °C, 16 °C, and 24 °C with non-stored control cell sheets.

## Results

**Figure 1:**
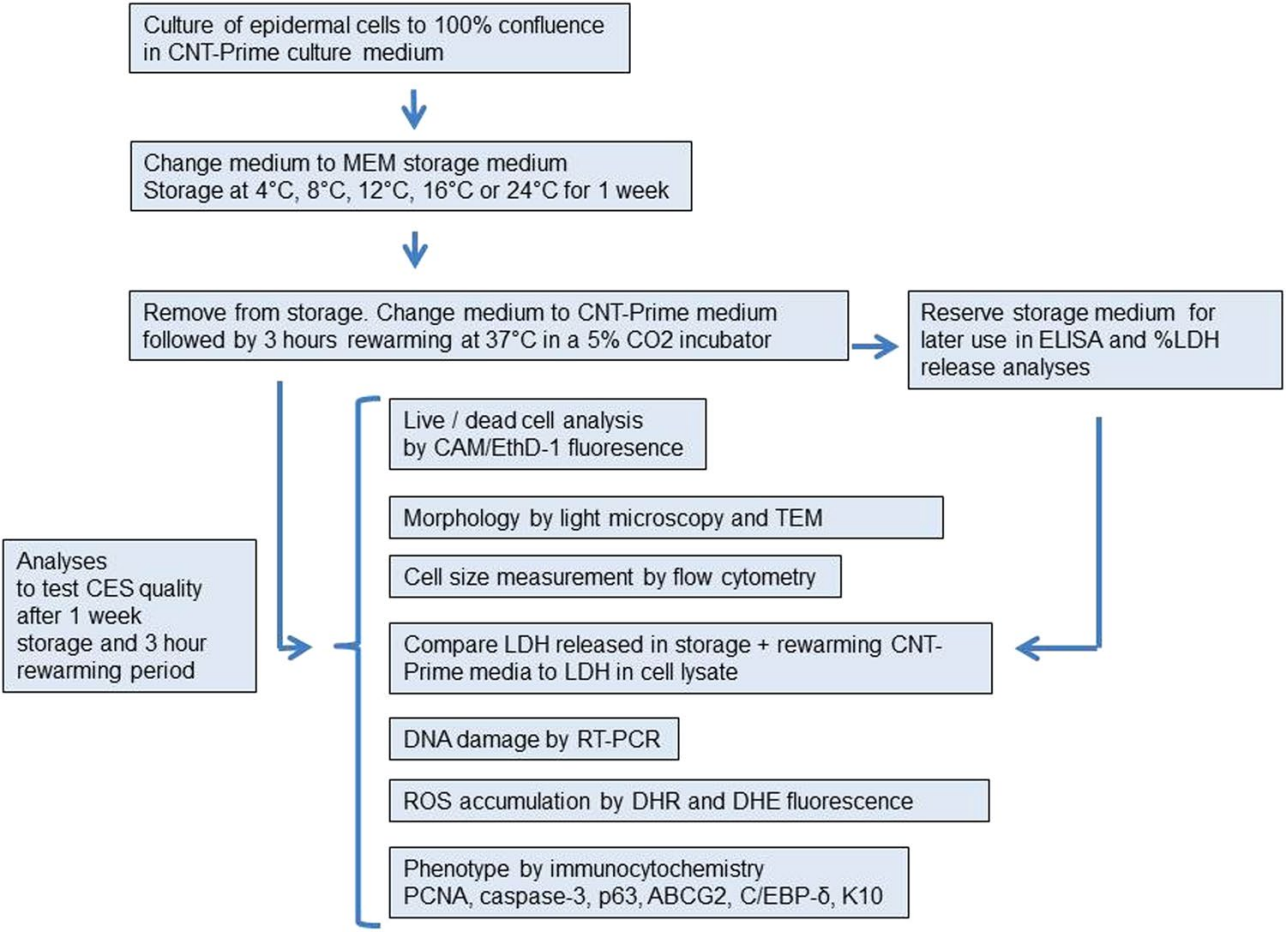
Workflow of culture, storage and quality-testing analyses.

Work flow is presented in Fig. 1.

### Viability and Cell Integrity

#### Storage Temperatures 12 °C and 16 °C were Optimal for Preservation of Viable Cells

**Figure 2:**
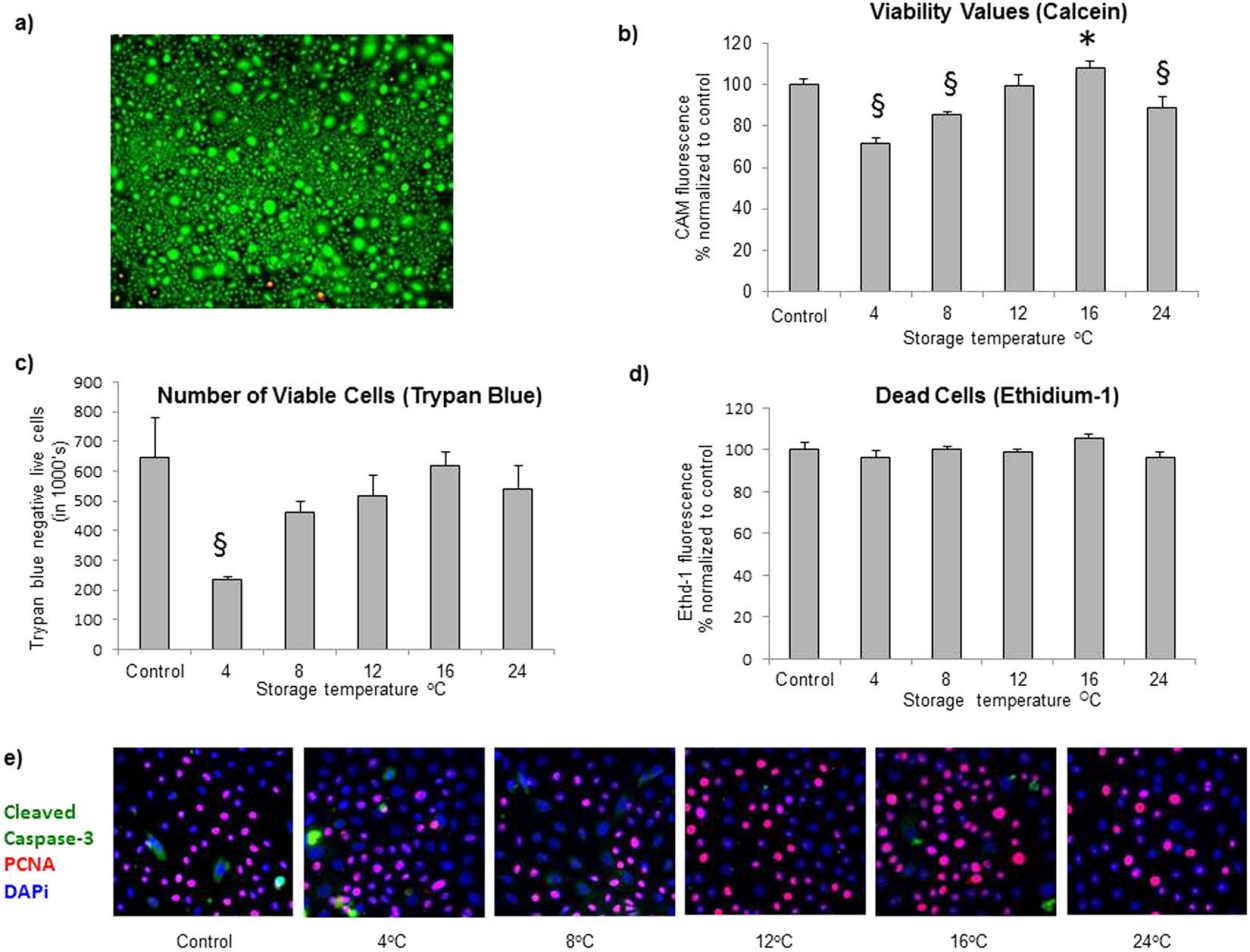
Viable and dead cells. (**a**) Representative live (CAM)/dead (EthD-1) fluorescence image in control cells (X40). The fluorescence value from the live (**b**) and dead (**c**) cells in control are represented as 100% in graphs for comparison with temperature groups. (**b**) The number of live cells, indicated by CAM fluorescence (^*^=significantly increased compared to control; ^§^=significantly decreased compared to control; *p* ≤ 0.05). (**c**) The number of dead cells, indicated by EthD-1 fluorescence. (**d**) The number of viable cells using trypan blue (^§^=significantly decreased compared to control; *p* ≤ 0.05) (**e**) Expression of PCNA and activated Caspase-3.

The number of live cells in stored temperature groups was compared to non-stored control by measurement of calcein acetoxymethyl (CAM) fluorescence and trypan blue (Fig. 2a–c). CAM fluorescence measures esterase activity inside the cell, whereas lack of intracellular blue dye staining indicates live cells when trypan blue is used. The highest percentage of live cells compared to the non-stored control was seen at 12 °C (99 ± 3%; *p* = 0.70) and 16 °C (108 ± 2%; *p* = 0.012) using CAM fluorescence (Fig. 2b and Table S1). All other temperatures had significantly lower live cell values compared to control (*p* ≤ 0.05). Using trypan blue to assess the number of living cells largely confirmed the same general trend of increasing viability with temperature until 24 °C (Fig. 2c). Trypan blue gave a significantly lower number of viable cells at 4 °C compared to non-stored control, with 237 ± 6.0 × 10^3^ cells, compared to control with 647 ± 133.2 × 10^3^ cells (p ≤ 0.05). The number of dead cells was assessed using ethidium homodimer-1 (EthD-1) fluorescence. No significant difference in dead cells was seen in any group compared to control (*p* ≥ 0.768) (Fig. 2d).

#### Optimal Preservation of Cell Proliferation was Observed at Storage Temperatures 12 °C and 16 °C

The nuclear protein PCNA is a marker for cell proliferation^19^. The percentage of cells positive for PCNA was significantly increased at 12 °C and 16 °C, with 58 ± 6% and 72 ± 5% respectively, compared to 40 ± 3% in control (*p* ≤ 0.05; Fig. 2e and Supplementary S1). High cell proliferation values at these temperatures corresponded to significantly higher cell viability (CAM fluorescence). All other groups had PCNA values comparable to control. Apoptotic cells were assessed by immunocytochemistry, using an antibody against activated caspase-3. Cleaved caspase-3 levels were stable throughout the temperature range, indicating no significant increase in apoptosis in any group compared to control (Fig. 2e and Supplementary S1). This aligned with analysis of cell death by EthD-1, but dead cells that had detached during storage were not accounted for by either of these methods (Fig. 2d and Supplementary S1) (minimum *p* = 0.064 at 4 °C).

#### Storage Temperature Influenced the level of Intracellular Oxidative Stress

**Figure 3:**
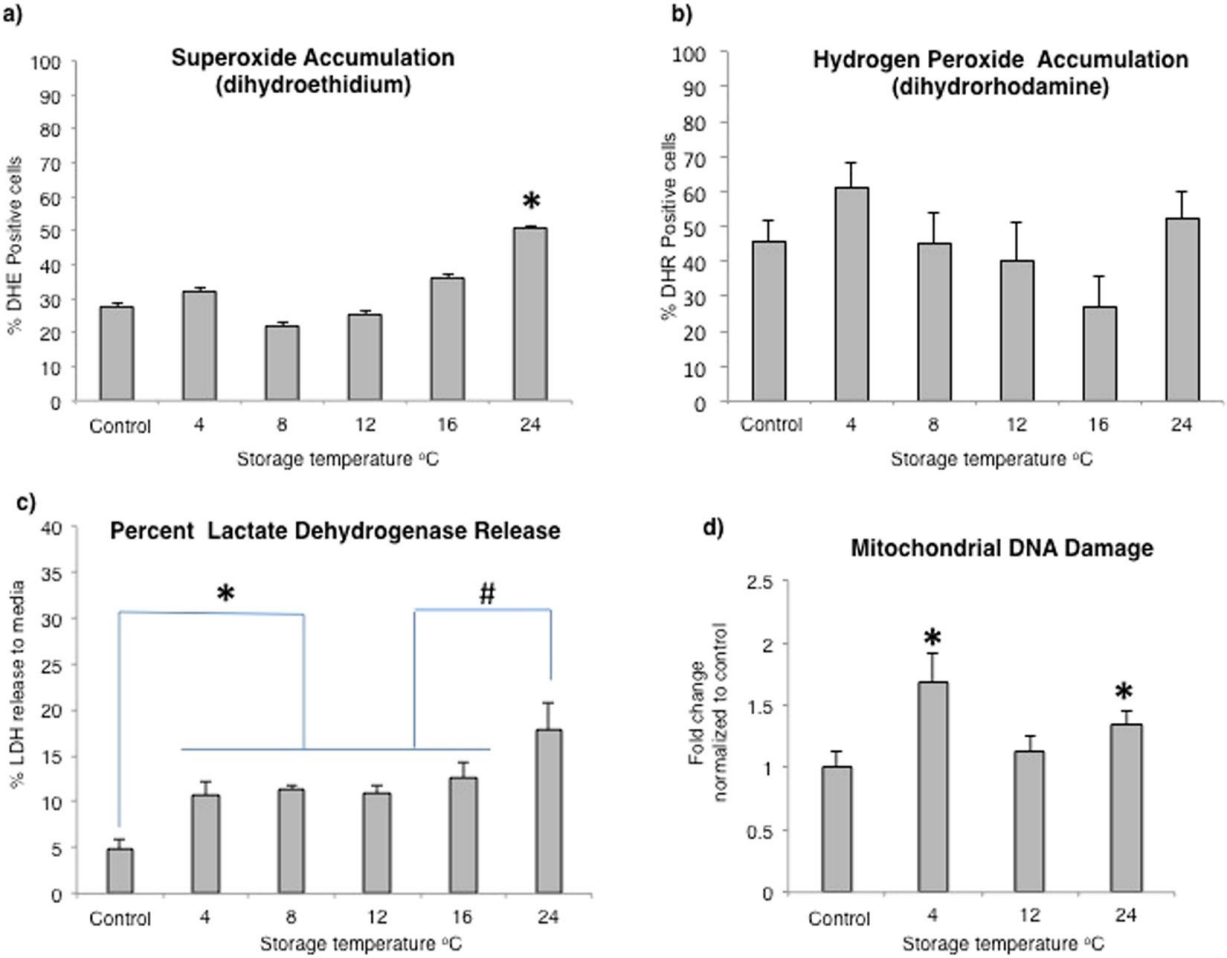
Mitochondrial reactive oxygen species and damage to macromolecules. (**a**) Mitochondrial superoxide anion level as indicated by dihydroethidium fluorescence (^*^=significantly increased compared to control; *p* ≤ 0.05). (**b**) Mitochondrial peroxyl levels as indicated by dihydrorhodamine fluorescence compared to control. (**c**) Membrane damage, indicated by percent lactate dehydrogenase release (^*^=significantly increased compared to control; ^#^=significantly increased compared to other temperature groups; p ≤ 0.05). (**d**) Fold change mtDNA damage (^*^=significantly increased compared to control; *p* ≤ 0.05).

Increased level of reactive oxygen species (ROS) such as hydrogen peroxide (H_2_O_2_), superoxide anion (·O_2_^−^), and hydroxyl (·OH^−^) strongly correlate with decreased cell viability^20^. Commercially available probes with distinct affinity for different types of ROS can be used to characterize the intracellular redox landscape. The probe dihydroethidium (DHE) enters cells freely and detects mitochondrial superoxide^21^. Only CES stored at 24 °C showed a significant increase in DHE fluorescence to 51 ± 4% indicating an increase in superoxide radical formation compared to control (26 ± 2%) (*p* ≤ 0.05) (Fig. 3a). The cationic probe dihydrorhodamine (DHR) is readily oxidized by mitochondrial peroxyl moieties, such as H_2_O_2_, and preferentially accumulates in mitochondrial compartments. No significant difference in DHR oxidation was seen between the groups or compared to control (Fig. 3b). Thus, the pattern of DHR oxidation had low correlation with mitochondrial superoxide.

#### Highest Increase in Cell Membrane Damage Was Seen at 24 °C

The lactate dehydrogenase (LDH) release assay gives an indication of cell membrane porosity and cell damage by quantifying the amount of LDH released to the media as a percentage of the total LDH (intracellular and extracellular). Cell damage was significantly increased at every temperature compared to control where the percent of LDH release was ~5% (*p* ≤ 0.05) (Fig. 3c). Release at 24 °C was significantly higher than at all other temperatures with ~17% damaged cells compared to ~12% at 4 °C, 8 °C, 12 °C and 16 °C (*p* ≤ 0.05).

#### Integrity of Mitochondrial DNA Was Maintained in Cells Stored at 12 °C

Mitochondrial DNA (mtDNA) is vulnerable to oxidation damage caused by intracellular oxidative stress. Hence, the mtDNA damage level is frequently used as a measure of oxidative stress. To investigate if the trend towards lower ROS accumulation at 12 °C, and higher levels at 4 °C and 24 °C could have impacted the integrity of intracellular macromolecules, mtDNA was assessed at these temperatures. Damage at 4 °C and 24 °C was significantly increased compared to control, with a fold change of 1.7 and 1.4 respectively (*p* ≤ 0.01), while no significant difference was detected at 12 °C compared to control (*p* = 0.24) (Fig. 3d).

Viability and cell integrity studies suggested that 12 °C is the optimum temperature for one-week storage of CES.

### Morphology

#### Cell Morphology Was Significantly Impacted by Storage Temperature

**Figure 4:**
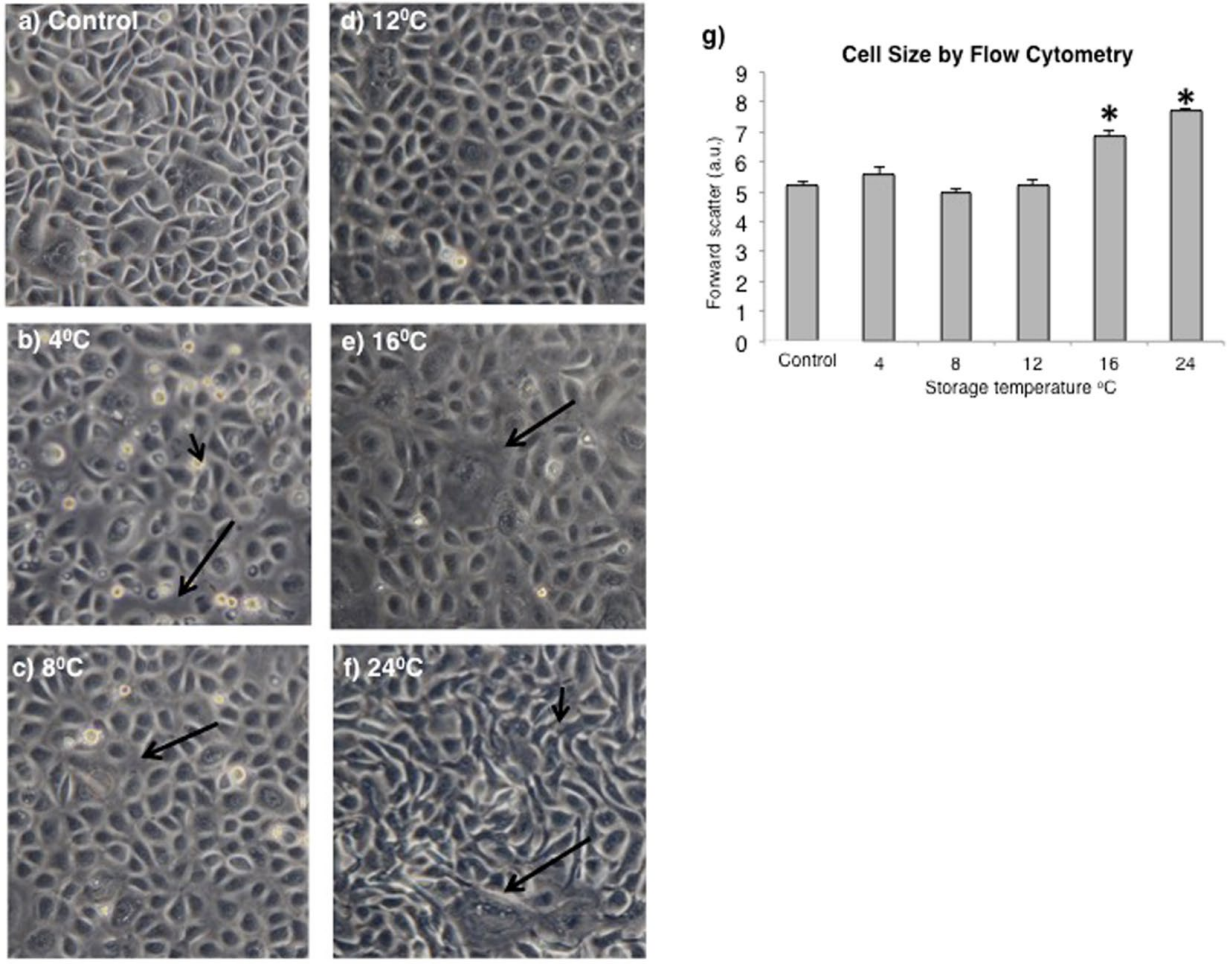
Cell morphology as shown by phase contrast (X200) micrographs. (**a**) Representative image of control. Gaps in the cell layer (arrows) were seen at 4 °C (**b**) and at 8 °C (**c**). Dead cells (arrowhead) were also present at 4 °C (**b**). The cell layer was similar to control at 12 °C (**d**). Large cells (arrows) were more frequent at 16 °C (**e**) and at 24 °C (**f**) Extended filopodia (arrowhead) were also seen at 24 °C (**g**) Cell size indicated by forward scatter by flow cytometry. (^*^=significantly increased compared to control; *p* ≤ 0.05).

Phase contrast microscopy was performed to investigate the effect of storage temperature on cell structure and ultrastructure. Analysis of microscopy results revealed that the non-stored CES control exhibited typical CES morphology, with a mosaic arrangement of small and some larger cells (Fig. 4a). Cell morphology after storage at 12 °C demonstrated an uncompromised CES (Fig. 4d), whereas at 4 °C and 8 °C, gaps in the cell layer were seen (arrows, Fig. 4b and c) and dead cells were present (arrowhead, Fig. 4b). Cell morphology at 24 °C was markedly different compared to control. Extended filopodia suggested many cells were stressed (arrowhead, Fig. 4f), and a higher fraction of large flat cells indicated differentiation (arrow, Fig. 4f). The 16 °C group also had more large-sized cells suggesting increased differentiation (arrow, Fig. 4e), but without elongated filopodia. Microscopy indicated that cell morphology at 12 °C was most similar to control and a complete cell layer was retained.

#### Frequency of Large-sized Cells Increased at 16 °C and 24 °C

Flow cytometry was used to assess cell size (Fig. 4g). Large cell size is characteristic of differentiated epidermal keratinocytes, whereas small cell size is associated with stem cells. Light scattered in a forward direction as a cell passes through the laser beam is related to the size of the cell^22^. Light travels further from a larger cell and thus forward scatter can be used to indicate cell size. Forward scatter measurements showed that cells in the lower temperature range, from 4 °C to 12 °C, maintained a mean cell size similar to control, whereas the average size of cells stored at 16 °C and 24 °C was significantly increased (*p* ≤ 0.05; Fig. 4g).

#### Transmission Electron Microscopy Indicated Differentiation at 24 °C

**Figure 5:**
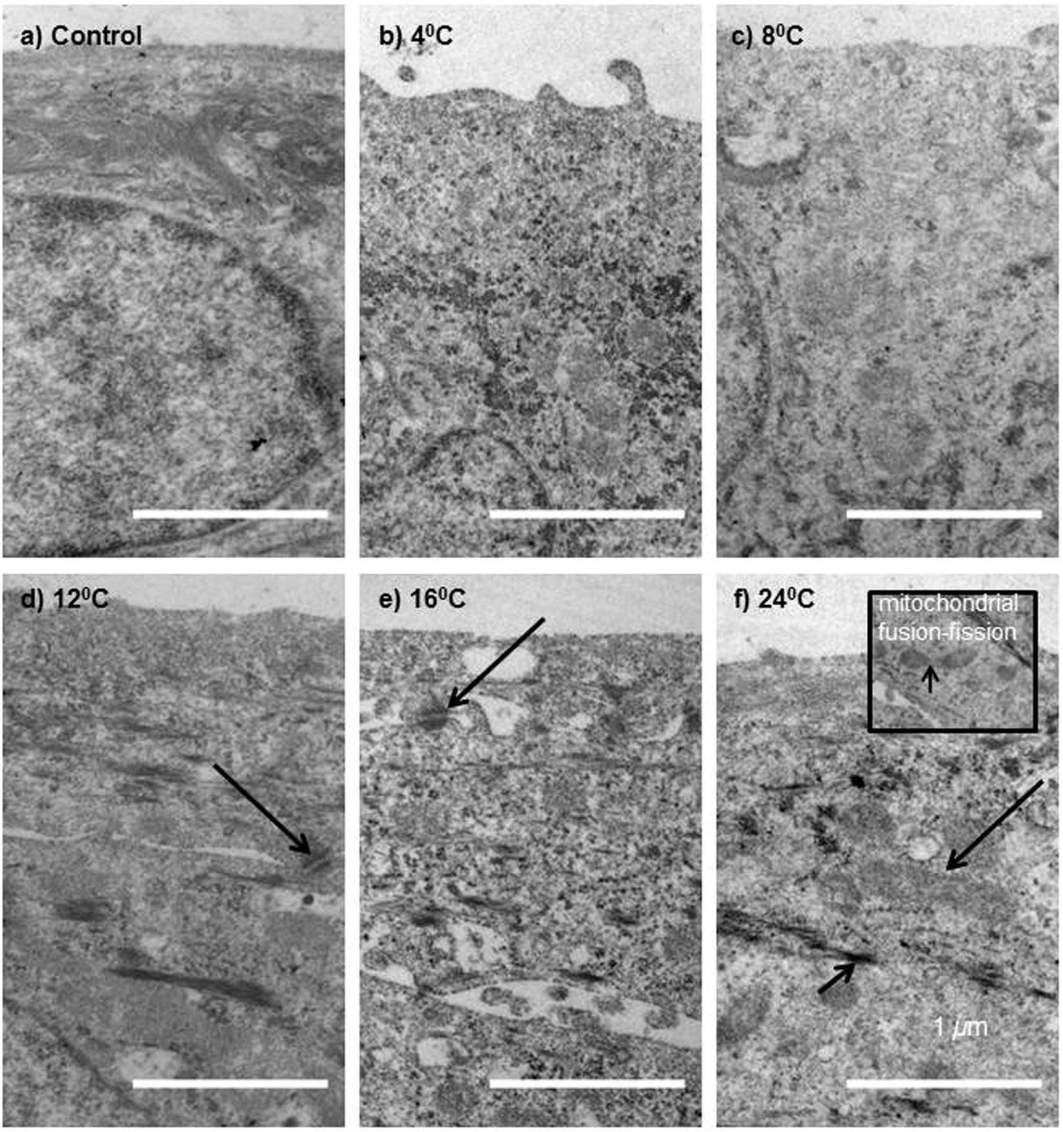
Cell morphology as shown by transmission electron microscopy (X24500) micrographs. Normal desmosome connections between cells (arrows) were seen at all temperatures, represented in (**e**) and (**f**). Large mitochondria (arrow) were more frequent at 24 °C (**f**). Mitochondrial fission or fusion (arrow) was also seen at 24 °C (inset (**f**)). Keratin bundles (arrowhead) were visible at all temperatures, but were more pronounced at 24 °C (**f**).

Transmission electron microscopy (TEM) observations showed normal desmosome connections between cells at all temperatures (arrows, Fig. 5d and e). There were signs of mitochondrial fission^23, 24^ or fusion at 24 °C (black arrow, insert Fig. 5f) and larger mitochondria were more frequent at 24 °C (Fig. 5f). Keratin bundles^25^ were visible at all temperatures but were more pronounced at 24 °C suggesting increased differentiation (arrow head, Fig. 5f).

Cell morphology studies using TEM suggested that 12 °C is optimal for storage of CES.

### Phenotype

#### The Storage Temperature 12 °C Demonstrated Highest Preservation of Undifferentiated Cell Phenotype

**Figure 6:**
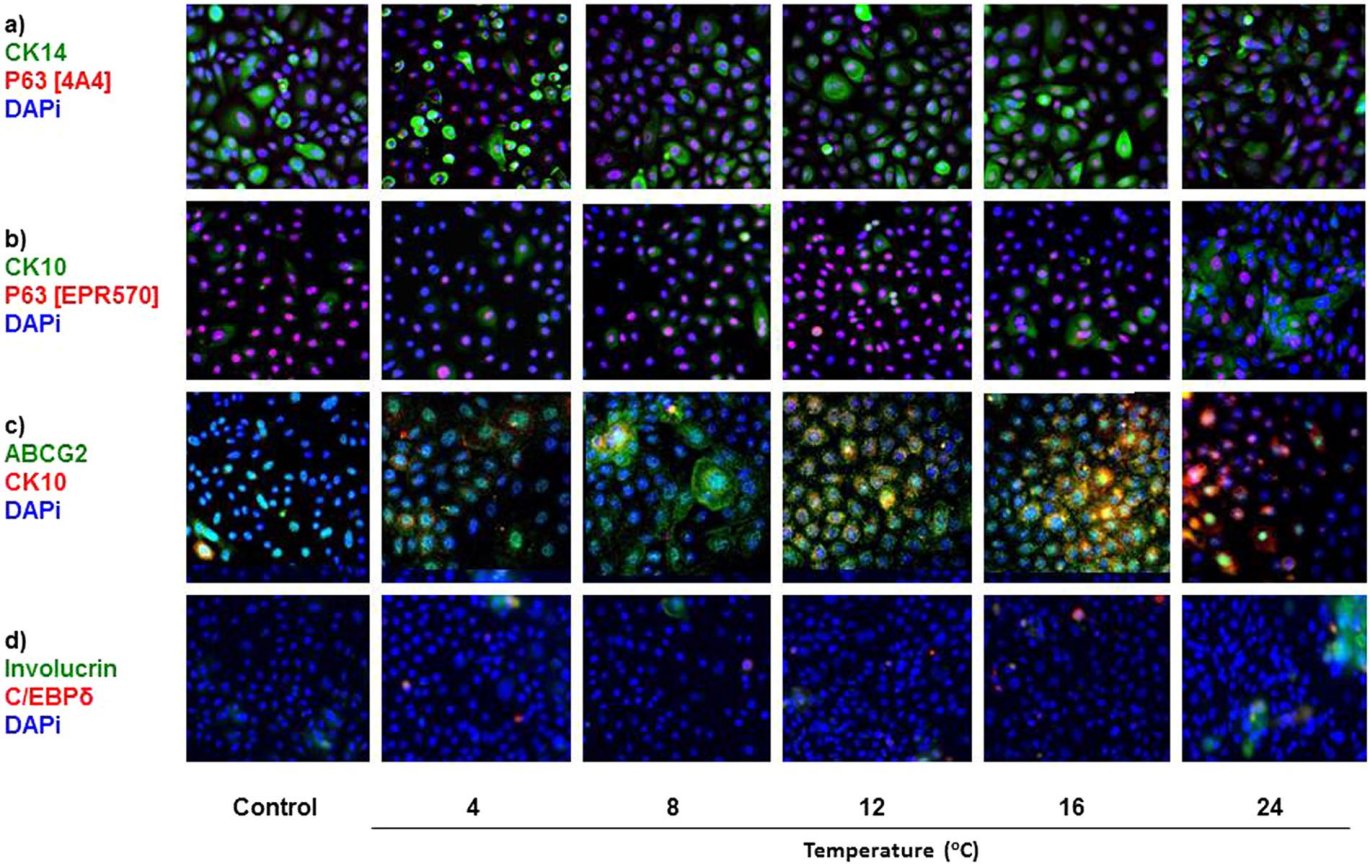
Immunocytochemistry using putative epidermal stem cell and differentiated epidermal cell markers. Figures show comparison of non-stored control with storage temperature groups. Magnification: 200X.

The markers tumor protein p63 (p63)^26^ and ABC transporter family G2 (ABCG2)^27, 28^ are associated with an undifferentiated cell phenotype, desirable for transplantation, while the transcription factor CCAAT/enhancer-binding protein (C/EBPδ)^29, 30^, involucrin and cytokeratin 10 (CK10)^31^ are markers of differentiated epidermal cells. P63 was used in combination with different keratin-specific antibodies; p63 [4A4 clone]/CK14 (CK14 = basal cells) and p63 [EPR5701 clone]/CK10 (CK10 = early differentiated cells) (Fig. 6a,b, Supplementary S1 and Table 1). Expression of p63 [4A4 clone] was only maintained similar to control (91 ± 2%) at 12 °C (92 ± 2%; *p* = 0.801) and 16 °C (97 ± 1%; *p* = 0.166) (Fig. 6a,b, Supplementary S1; Table 1). Closer analysis of p63 in cells disaggregated from the cell sheet using the cytospin technique confirmed that p63 expression was significantly lower than control at 4 °C and 24 °C (*p* ≤ 0.05) (Figure Supplementary S2). CK14 followed a similar expression pattern to p63 [4A4 clone]; weaker expression was seen at 24 °C. These results indicated that 12 °C and 16 °C were the best temperatures for retention of undifferentiated cell phenotype during storage.

**Table 1:** Percent Positive Cells (Immunocytochemistry).

	Control	4 °C	8 °C	12 °C	16 °C	24 °C
ABCG2 [BXP21]	+++	++++	+++++*	+++++*	+++++*	++*
CK10 [polyclonal]	+	++	++	++	+++*	+++*
P63 [EPR5701]	+++++	++++*	++++*	+++++	+++++	++*
P63 [4A4]	+++++	++++*	++++*	+++++	+++++	++*
CK14 [LL002]	+++++	+++*	+++++*	+++++	+++++	++++*
Involucrin [SY5]	+	+	+	+	+	++*
C/EBPδ [polyclonal]	+	+	+	+	++*	+
PCNA [PC10]	+++	+++	+++	+++*	++++*	+++
Cleaved Caspase-3 [D175]	+	+	+	+	+	+

^*^Significantly different compared to control (p ≤ 0.05). + = present. ++ = 20–39%. +++ = 40–59%. ++++ = 60–79%. +++++ = 80–100%.

Expression level and localization of ABCG2, a heme and multidrug transporter, varied considerably between temperature groups (Figs. 6c, S1; Table 1). In control conditions, ABCG2 fluorescence was shown in 59 ± 6% of cells and mostly localized to the nucleus. The expression level was not significantly changed at 4 °C, but the pattern of expression included perinuclear, nuclear, and cell membrane localization. A significantly increased number of cells with ABCG2 fluorescence was seen in the temperature groups 8 °C, 12 °C and 16 °C, where all were ~80% ABCG2 positive. At these three temperatures, the transporter appeared to be predominantly localized to the perinuclear region, with some expression at the cell membrane and nucleus. The most intense perinuclear staining for ABCG2 was in a punctate pattern at 12 °C and 16 °C. Significantly reduced ABCG2 expression, mostly at the nucleus, was seen at 24 °C, with only 27 ± 5% (*p* ≤ 0.05) positive staining of stored cells (Figs. 6c, S1; Table 1). According to ABCG2 expression, the storage temperatures 8 °C, 12 °C and 16 °C were best for storage of CES.

The cytokeratin CK10 is found *in vivo* in suprabasal epidermal cells, and is first expressed in early differentiation^31^. All CK10 expressing cells also expressed ABCG2 except at 24 °C, where the percentage of cells expressing CK10 was significantly higher, with 59 ± 14%, and ABCG2 fluorescence lower. The number of CK10 positive cells increased at 16 °C and 24 °C with 56 ± 11% and 59 ± 14% staining respectively, compared to control at 10 ± 2% (*p* ≤ 0.05) (Fig. 6c, Supplementary S1; Table 1). Expression of C/EBPδ was seen in the cytoplasm of isolated cells in control with 5 ± 1% (Fig. 6d). Expression was similar to control at all temperatures apart from at 16 °C with significantly increased expression (27 ± 11%; *p* ≤ 0.05). Though C/EBPδ is a transcription factor, expression was seen in the nucleus and cytoplasm. Increased C/EBPδ expression at 16 °C suggested onset of differentiation. Therefore, 16 °C is less suitable for storage of CES.

Involucrin also showed significantly increased expression at 24 °C with 21 ± 17% compared to control at 3 ± 1% (*p* ≤ 0.05). Involucrin expression occurred in clusters at 24 °C (Fig. 6d, Supplementary S1; Table 1). Increased expression of CK10 at 16 °C and 24 °C, as well as increased involucrin at 24 °C indicated more differentiation and suggested that these storage temperatures were less suitable for optimum storage of CES.

These phenotype studies showed that 12 °C was the best storage temperature as undifferentiated phenotype was best maintained at this temperature compared to control.

### Cytokine Secretion

#### Media Analyses Showed Secretion of Cytokines Relevant to Regeneration of Epithelia

**Figure 7:**
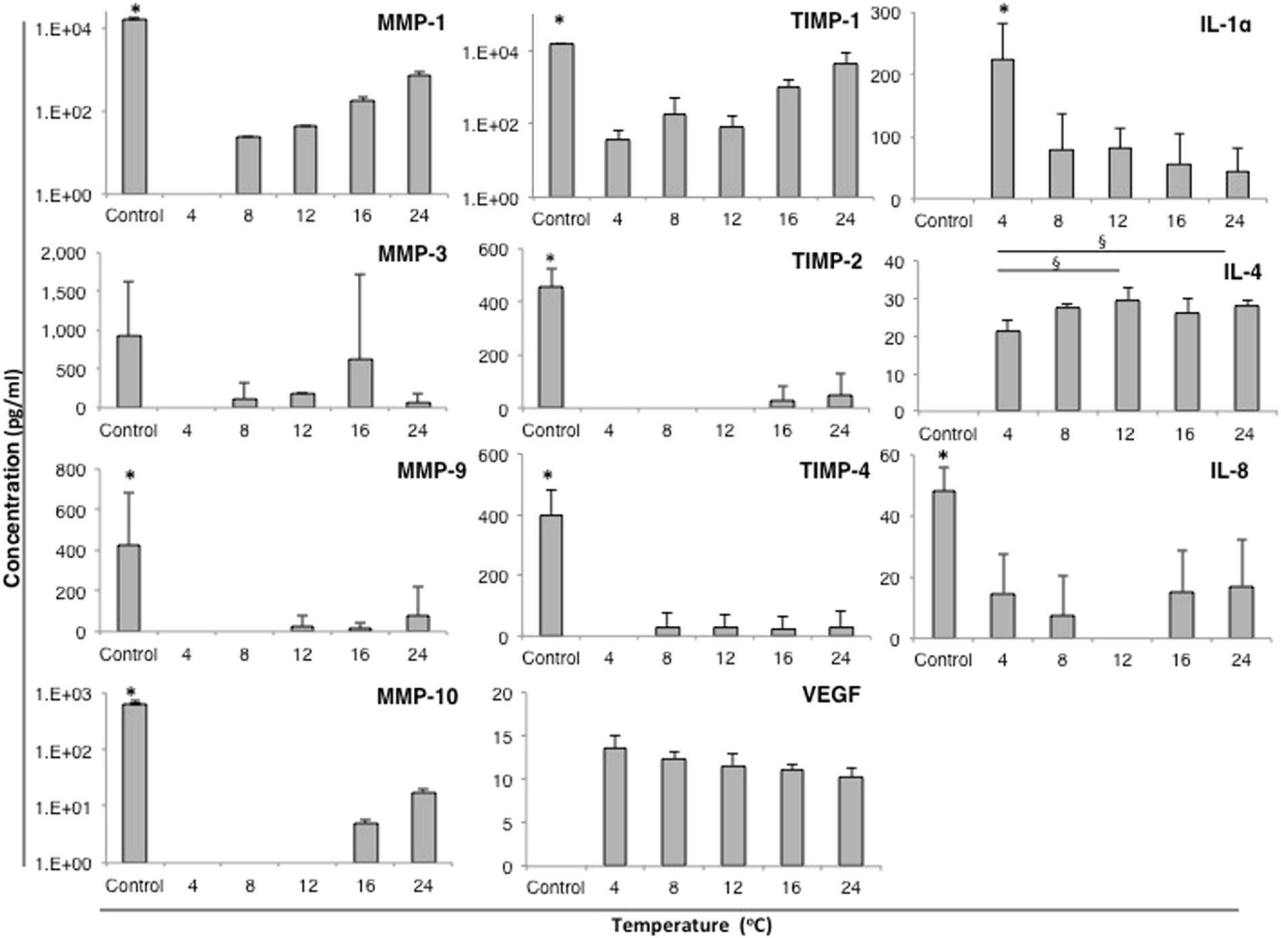
Cytokines and growth factors measured in medium from cultured cells before storage and in storage medium following one-week storage. For comparison of secretion during storage, figures show non-stored control cell secretion after subtraction of CNT-Prime culture medium alone (without cells). IL = interleukin; MMP = matrix metalloproteinase; TIMP = tissue inhibitor of matrix metalloproteinase; VEGF = vascular endothelial growth factor; (^*^=significantly increased; ^§^=significantly decreased; *p* ≤ 0.05).

To give an indication of the regenerative potential of cultured sheets following one-week storage we tested media for secreted growth factors and cytokines relevant to wound healing using enzyme-linked immunosorbent assays (ELISAs). The following cytokines were analyzed: interleukins (IL) IL-1α, IL-1β, IL-4 and IL-8; transforming growth factor (TGF-β1 and TGF-β3); vascular endothelial growth factor (VEGF); matrix metalloproteinases (MMPs) 1, 2, 3, 8, 9, 10 and 13 and the tissue inhibitors of metalloproteinases (TIMPs) 1, 2 and 4. The cytokines TGF-β1, TGF- β3, EGF, MMP2, MMP8 and MMP13 were not found in any groups before or following one-week storage (data not shown). Subtraction of values for CNT-Prime medium alone (without cells) from the controls showed that IL-1α, IL-4 and VEGF are not normally secreted by CES under our culture conditions (Fig. 7; Supplementary S3). However, these cytokines were up-regulated during one-week storage at all temperatures. In contrast, IL-8, MMP-1, MMP-9, MMP-10, TIMP-1, TIMP-2 and TIMP-4 levels were significantly higher in non-stored control medium compared to storage media from all temperature groups (Fig. 7; Supplementary S3). The cytokines MMP-1, MMP-9, MMP-10, TIMP-1 and TIMP-2 showed a tendency to increase with storage temperature, while IL-1α and VEGF tended to decrease at higher temperatures, though these tendencies were not significant. IL-1α was significantly increased at 4 °C compared to all other temperatures (p < 0.05), whereas IL-4 was significantly decreased at this temperature compared to 12 °C and 24 °C (p < 0.05). Overall there was a large variation in regulation of individual cytokines in response to storage.

## Discussion

Retention of undifferentiated cell phenotype in cultured and stored CES is important for the treatment of patients with burns^10, 12^. Based on recent findings an undifferentiated cell phenotype is also likely to be important in the regenerative treatment of other epithelia using CES^4, 5, 11^. We show here that choice of storage temperature has a profound effect on the integrity and phenotype of stored CES. Morphology and phenotype analyses revealed that CES was most differentiated at 16 °C and 24 °C and therefore less suitable for transplantation. While retention of a high percentage of live cells was seen at 12 °C and 16 °C, storage at 12 °C uniquely provided optimal morphology and undifferentiated phenotype. Onset of differentiation at higher temperatures may be related to accumulation of ROS associated with the predictable increase in metabolic activity^24^.

Maintenance of cell viability is the first priority in storage of CES transplants. Cell viability measured by CAM fluorescence was 70% or greater in all temperature groups when normalized to control. Assessment of the number of viable cells by trypan blue also showed the same trend. The slight differences between the two methods might be explained by the more qualitative estimate of trypan blue exclusion versus quantification of fluorescent signal emitted by CAM. Viability was improved compared to results in our two-week storage study^17^ where cell viability values in temperature groups between 4 °C and 37 °C typically ranged between ~20% and ~60%. Cell viability was comparable at 24 °C between the two studies (~90% normalized to control). This infers that CES are less stable over a longer period at lower temperatures, whereas increased differentiation may have contributed to improved stability over a longer time period at 24 °C^17^. It is interesting to note that while 16 °C had a high number of viable cells following storage for one week, we reported only 30% viability at this temperature following two-week storage^17^. This may be explained by 16 °C representing the critical point at which differentiation signaling is activated in combination with accumulating damage associated with ongoing metabolism. It could be that during one-week storage at 16 °C CES continue to experience signals to differentiate, but are not at a warm enough temperature to allow full differentiation. In contrast, at 24 °C cell sheets are able to fully differentiate, which provides stability over a longer two-week timeframe.

Apoptosis shown by caspase-3 cleavage and cell death as demonstrated by EthD-1 fluorescence were minimal at all temperatures compared to control. However, a caveat is that more dead cells had detached at 4 °C and 8 °C and were therefore not included in analyses. Highest viability at 12 °C and 16 °C corresponded to significantly increased PCNA expression, potentially^32^ demonstrating the importance of actively cycling cells in maintenance of CES viability. Overall, our results align with a study by Oie Y. *et al*. in 2014, where viability after transport control was shown to increase by ~4% following a 12 hour transport period at above-freezing temperature^33^. Our cell viability, cell death, PCNA, and Caspase-3 results indicated optimal maintenance of CES viability at 12 °C and 16 °C following one-week storage.

Accumulation of ROS can cause potential long-term oxidation damage to macromolecules, such as lipids and DNA^20^. Generation of ROS may therefore have been the primary cause of membrane damage, which was significantly increased in all temperature groups, but was especially high at 24 °C, as shown by lactate dehydrogenase enzyme release. Although more experiments are needed to address the source of ROS during storage, the preferential oxidation of superoxide-sensitive DHE suggests that superoxide production in mitochondria could be a factor limiting storage viability of CES.

In accordance with findings by Keyer and Imlay, superoxide may be responsible for damage to mtDNA via the release of divalent iron from iron sulfur-clusters in the mitochondria^34^. Divalent iron and dismutated superoxide (to peroxide) produce the highly reactive hydroxyl radical that damage mtDNA. In alignment with generation of superoxide species, the present study showed mtDNA damage was minimal at 12 °C, but significantly increased at 4 °C and 24 °C. These results suggest that inhibition of mitochondrial superoxide production could be a fruitful strategy to increase longevity of stored CES. Results were similar to findings by Zieger *et al*. who demonstrated 10 °C was the optimum temperature for storage of cultured endothelial cells due to minimal damage from oxidative stress^35^. The damage sustained by membrane lipids and metabolic enzymes may have led to further ROS generation and a reinforcing cycle of accumulating damage to mtDNA^36^. Together, these results suggest that viability and cell integrity were best maintained in the mid-range of the temperature groups, centered at 12 °C, whereas cells stored at lower and higher temperatures, 4 °C and 24 °C, accumulated most damage, potentially due to increased ROS generation.

Phase contrast and TEM observations corroborated cell viability and cell damage assessments. Many cells had detached at lower temperatures, leaving a compromised CES layer. In agreement with our two-week storage study^17^, optimal morphology and ultrastructure were conserved similar to control at 12 °C. Increased cell size shown by flow cytometry and phase contrast observations, and prominent keratin bundles shown by TEM, indicated more differentiation at 16 °C and 24 °C^37^. Adaption to temperature could reflect not only the level of damage from cold-inflicted stress, but also the capacity of cells to respond to ROS, based on enzymatic function and cell readiness for onset of differentiation^24^.

Maintained expression of the progenitor cell marker p63 suggested that retention of undifferentiated cell phenotype was best at 12 °C and 16 °C. The marker for basal keratinocytes, CK14, was widely expressed in all groups except at 24 °C, where expression decreased. CK10 was significantly increased at 16 °C and 24 °C. Some cells were positive for p63 and CK10. These were seen in all storage temperature groups as well as in the control and could suggest transition from transient amplifying (TA) phenotype to early differentiation. C/EBPδ, a transcription factor that works in concert with p63 in epidermal cells to initiate differentiation^30^ was also seen in all groups but was significantly increased at 16 °C. Expression patterns of p63, CK14, C/EBPδ and CK10 combined with morphology observations, supported retention of undifferentiated cell phenotype at 12 °C, transition to differentiation at 16 °C, and increased differentiation at 24 °C. These observations are in agreement with our two-week study^18^ indicating a more differentiated phenotype at 24 °C compared to 12 °C. However, at colder temperatures, 4 °C and 8 °C, C/EBPδ and CK10 fluorescence was lower following one-week storage compared to two-week storage, suggesting that cold-inflicted stress may have a cumulative impact on gene expression over a longer period.

Since *abcg2* expression is regulated by hypoxia-inducible factor-1α ﻿(HIF-1α)^38^, adaption of cells stored at different temperatures may be partly explained by the protection afforded by this transporter through management of ROS generation^24^. ABCG2 fluorescence in the cytoplasm and perinuclear sub-compartments as seen at 12 °C and 16 °C has been similarly reported by others^39, 40^. This raises the speculation of whether ROS signaling, generated in mitochondria^41^, results in increased *abcg2* transcription through a feedback mechanism dependent on stabilization of HIF-1α by ROS^42^. The contribution of ABCG2 to management of ROS may be through its established role in transport of heme and porphyrins^38^. These iron-containing complexes can provide divalent iron for Fenton reaction-mediated hydroxyl radical generation. Thus, in addition to its identified role in stem cells, efflux or sequestration of heme to intracellular compartments by ABCG2 could protect cells. This novel role may be especially relevant to epidermal keratinocytes, where progression towards differentiation depends on a balance between apoptosis and proliferation, which is managed at the mitochondrial axis^23, 24^.

Surprisingly, we found nuclear localization of ABCG2 in the control and throughout the temperature range. This was less prominent in CES in our two-week storage study^18^, which could be due to the use of a different antibody clone (H-70 compared to BXP-21 used here). To our knowledge, nuclear expression of this transporter in CES has not been previously reported, though ABCG2 has been shown to act as a nuclear transcription factor in the transcription of E-cadherin in lung cancer cells^43^. Alternatively, nuclear membrane localization of ABCG2 would suggest a nuclear efflux role for this transporter in epidermal keratinocytes, as has been shown in other cell types^44–46^.

A recent report by Ma *et al*. used ABCG2 as a stem cell marker in the interfollicular epidermis, where stem cells accounted for 2.1–3.3% of total basal keratinocytes by flow cytometry^28^. In sharp contrast, we found that ABCG2 fluorescence was as high as ~90% at 12 °C and 16 °C, and some cells co-expressed CK10. Co-expression of these two markers has been shown in human skin sections by immunohistochemistry^47, 48^. Hashimoto *et al*. showed that ABCG2 is expressed throughout basal and supra-basal layers in human epidermis, and mostly coincides with CK10 expression. Expression of ABCG2 in suprabasal layers also corresponds to immunohistochemistry staining that we have noticed in sections of normal skin^18^. Similarly, Triel *et al*. showed that high ABCG2 expression in side population cells isolated by the Hoechst dye flow cytometry assay identifies TA cells^48^. Thus, it is possible that high ABCG2 expression at 12 °C and 16 °C identifies a large population of highly proliferating TA cells as well as progenitor cells^49^. The coincidence with high PCNA expression at these temperatures also supports this. In contrast, at 24 °C, ABCG2 fluorescence dropped significantly to 27 ± 5%, suggesting that expression of this transporter was redundant in cells at a later stage of differentiation^27, 49^. This was reflected by a similar value at 24 °C after two-week storage (39 ± 3%)^18^. However, a relative decrease in ABCG2 expression was seen at 12 °C and 16 °C after two-week storage, compared to one-week storage, which could reflect a decline in the number of TA cells with prolonged exposure to storage conditions^18^.

Media ELISA tests revealed that several cytokines important to wound healing were accumulated over the one-week period. Secretion of MMPs and their inhibitors is tightly regulated, both spatially and temporally, to orchestrate normal wound healing^50^. It is therefore important that CES maintain this capacity during storage. MMP-1, which is important in keratinocyte migration^51^ and re-epithelialization through remodeling of the extracellular matrix^52^, was secreted at 8, 12, 16 and 24 °C. The MMP-1 inhibitor, TIMP-1, was also expressed at these temperatures and both showed a tendency to increase with temperature. Of special interest were IL-1α, IL-4 and VEGF that were not expressed in non-stored control. Secretion of IL-1α, a cytokine involved in skin barrier formation^53^ collagen secretion^54^ and keratinocyte migration^55^, was significantly higher at 4 °C. This may suggest its function in protection of skin against cold. Secretion of IL-4 and VEGF was seen at all temperatures, with highest IL-4 expression at 12 °C and 24 °C. These factors are noteworthy for their established association with skin repair through stimulation of angiogenesis^56^. Overall, we found that cytokines secreted by non-stored control CES were also present at a lower level in stored cell sheets despite the larger volume of storage media. In addition, IL-1α, IL-4 and VEGF, which are significant factors involved in wound healing, were newly induced in stored CES.

All experimental groups contained identically cultivated cell sheets at the start of one-week storage. It is therefore remarkable to observe the radical differences in viability, morphology, and phenotype sustained in response to variation in storage temperature. In the present study, we showed that choice of storage temperature is critical. Storage of CES at 12 °C may improve the treatment of patients with burns and other skin injuries through maintenance of a high percentage of undifferentiated cells that have high proliferative capacity within the transplant^10, 12^. Likewise, retention of a high fraction of undifferentiated cells may be more conducive to cells responding to novel differentiation cues in the new environment when used for regenerative treatment of other types of epithelia. This has been illustrated by the successful integration of CES transplants in the treatment of LSCD and in the treatment of damaged urethral epithelium in animal models^4, 5^. Short-term storage at 12 °C has potential to expand the utility and increase access to CES in regenerative medicine by providing an option for transportation, increasing flexibility in scheduling surgery, and improving quality control. A limitation of the current work is the lack of animal studies that investigate successful integration of CES following storage in xenobiotic-free medium at 12 °C. This study prepares the way for such future work by providing analyses that represent a thorough characterization of stored sheets at each temperature. A future clinical trial might follow a similar design to that recently conducted by Vasania *et al.* ^57^, where cultured human conjunctival epithelial cells were transported to four regional centers from a centralized culture facility in India. Transplants were successfully used to treat patients with pterygium following transport^57^. A trial such as this might employ the optimal conditions outlined in the present study and transport CES for treatment of patients with chronic skin ulcers, which is an operation that can be scheduled in advance.

## Materials and Methods

Normal adult human epithelial keratinocytes (HEKa) were obtained from ScienCell Research Laboratories (San Diego, CA). The defined proprietary culture medium, CNT-Prime, was obtained from Cellntec Advanced Cell Systems AG (Bern). Goat serum, trypsin-ethylenediaminetetraacetic acid (EDTA), 4-(2-hydroxyethyl)-1-piperazineethanesulfonic acid (HEPES), sodium bicarbonate, sodium azide, Tween-20, Triton X-100, gentamicin, bovine serum albumin (BSA), fetal bovine serum (FBS), 4′,6-diamidino-2-phenylindole (DAPI), dispase II, soybean trypsin inhibitor, DHR, DHE, menadione and trypan blue were purchased from Sigma Aldrich (St Louis, MO). Nunclon Δ surface multidishes, glass coverslips, pipettes and other routine plastics were obtained from Thermo Fisher Scientific (Waltham, MA). Phosphate buffered saline (PBS), Hanks balanced salt solution (HBSS) and minimum essential medium (MEM) were from Life Technologies (Carlsbad, CA).

### Cell Isolation

After obtaining local ethical approval and informed consent in accordance with the Declaration of the Helsinki convention, epidermal keratinocytes were isolated from skin from a living donor. Experimental protocols for the isolation and use of epidermal keratinocytes were approved by the Regional Ethical Committee for Medicine and Health South-east Norway reference: 2013/815/REK South-east C. The donor was a 46-year old female undergoing abdomen reduction surgery. The sub-cutaneous adipose and most of the fibrous dermal tissue was removed, leaving a thin dermis and epidermal layer. Pieces ~2 cm × 0.3 cm were incubated in 1 ml of 1:1 dispase II + CNT-Prime medium with 100*u*g/ml penicillin and streptomycin antibiotics at 4 °C overnight. The epidermis was then separated from the dermis and incubated with trypsin (0.025% + EDTA) at 37 °C for 10 minutes. Trypsin was quickly neutralized using soybean trypsin inhibitor (1 mg/ml) at a 1:1 ratio. Primary cells were initially seeded at a density of 8000 cells/cm^2^, in serum-free CNT-Prime medium, on 60 cm^2^ dishes coated with 1 *µ*g/cm^2^ collagen IV, BD Biosciences (New Jersey, USA).

### Cell Culture

Epidermal keratinocytes at passage three from Sciencell (HEKa) or isolated from skin were seeded at 5000 cells/cm^2^ in serum-free CNT-Prime medium on multidishes coated with 1 *µ*g/cm^2^ collagen IV. Cells were cultured under normal conditions (5% CO_2_ and 95% humidified air at 37 °C). Culture medium was changed every two days. Upon confluency, cells were used for storage experiments. The experiment scheme for the study was executed as shown in Fig. 1.

### Cell Storage

Following culture, each multidish was sealed and randomly selected for storage at one of five different temperatures 4 °C, 8 °C, 12 °C, 16 °C, and 24 °C (n = 4 for each temperature). The standard deviation of the temperature in each storage container was ±0.4 °C as demonstrated previously^14^. The storage medium was MEM with 25 mM HEPES, 3.57 mM sodium bicarbonate and 50 *μ*g/ml gentamycin. Cultured HEKa, not subjected to storage, served as controls. Cells were stored for 7 days in air-tight culture wells sealed with Nunclon adhesive sheets. Following storage, MEM storage medium was replaced with CNT-Prime medium and cells were allowed to equilibrate in the 37 °C incubator for 3 hours before all analyses in order to assess any potential damage incurred upon rewarming^35^.

### Viability and Cell Integrity

#### Number of Live and Dead Cells

The number of live cells and cell death were assessed using a standard live/dead kit (Invitrogen Live/Dead Analysis Kit, Life Technologies, Grand Island, USA). Cells were incubated with CAM (2 *µ*M) and EthD-1 (1 *µ*M) (n = 4). After passing through the cell membrane, the ester group in CAM is cleaved by esterases in live cells to yield the membrane impermeable calcein green fluorescence. EthD-1 permeates the membrane of dead cells, and labels nucleic acids to yield red fluorescence. Cells were incubated at room temperature for 45 minutes. Fluorescence was measured with a microplate fluorometer (Fluoroskan Ascent, Thermo Scientific, Waltham, MA) with the excitation/emission filter pairs 485/538 nm for CAM and 530/620 for EthD-1. Background fluorescence, measured in wells containing CAM and EthD-1 without cells, was subtracted from all values. The values for live and dead cells in temperature groups were normalized to control values. Trypan Blue (0.4% solution) (Sigma) was used to quantify viable cells. Cells excluding dye were counted as live cells.

#### Analysis of Reactive Oxygen Species Using Flow Cytometry

Flow cytometric analysis of intracellular ROS was performed in separate analyses. DHR or DHE were added at a final concentration of 3 *µ*M to separate wells (n = 4 each). After incubation (5% CO_2_ and 95% humidified air) for 90 minutes at 37 °C (DHR) or for 20 minutes at 30 °C at ambient CO_2_ (DHE), cells were detached with 3 minutes trypsin incubation, washed and re-suspended in ice-cold HBSS + 4% FBS. Samples were kept on ice and analyzed using the BD Accuri C6 bench top flow cytometer. The production of ROS could be evaluated due to the transformation of the probes from non-fluorescent to fluorescent compounds by the oxidative burst intermediates within the cells. Excitation of the DHR dye at 488 nm produced green fluorescence with a peak at 530 nm, detected using the standard filter 530/30 (FL1). Excitation of the DHE dye at 488 nm produced red fluorescence with a peak at 600 nm, detected using the standard filter 585/40 nm (FL2). The percentage of cells positive for each probe was recorded for each experiment. Samples with no addition of probe (negative control) and samples pre-incubated for 30 minutes with 1 mM menadione (ROS generation positive control) were included.

#### Lactate Dehydrogenase Release Determined by Colorimetric Assay

LDH, a cytoplasmic enzyme, is released extracellularly when cell membranes are compromised^58^. Cell membrane damage was assessed using a colorimetric assay for LDH release to the media (LDH assay kit, Abcam, Cambridge, UK). Upon removal of cells from storage, the medium was collected and kept for subsequent analysis. Normal culture medium (collected after the normal three hour rewarming period) was added to the reserved storage medium. Cells were lysed in 1% Triton-X 100 HBSS. The LDH released to the collected storage and culture media and LDH in cells were measured separately using an LDH activity colorimetric assay kit (Sigma Aldrich, MO, USA), following the protocol recommended by the manufacturer. LDH was measured at 450 nm using a spectrophotometer and %LDH release was calculated (LDH in medium/(LDH in cells + LDH in medium) × 100%). Temperature groups were compared based on %LDH release relative to control (n = 4).

#### Mitochondrial DNA Integrity Assessed by Quantitative Real-Time Polymerase Chain Reaction

Mitochondrial DNA (mtDNA) integrity was assessed by the ability to inhibit restriction enzyme digestion, as reported previously (2015)^59^. Briefly, total DNA from control and cells stored at 4 °C, 12 °C, and 24 °C was isolated using the QiagenAmp Blood and Tissue Kit. Quantification and purity of DNA was measured using an Epoch Microplate Spectrophotometer (Bio-Tek). Primers designed for mitochondrial gene mt-Rnr1 (actcaaaggacttggcggta and agcccatttcttcccatttc) were used to analyze DNA damage by using two separate reactions (6 ng total DNA per reaction). The mtDNA damage was calculated from the difference in CT values in the group by the relation: damage frequency = 1/2exp(delta CT), and presented relative to that of non-stored cells ( = 1).

### Morphology

#### Light Microscopy Analysis

Light microscopy images were taken before and after storage at each temperature. Images were taken at random positions within each well at 200X magnification, using a Leica DM IL LED microscope and Canon EOS 5D mark II camera (Canon, Oslo, Norway). Individual cell morphology as well as the integrity of the complete cell layer were assessed.

#### Cell Size by Flow Cytometry

After exclusion of cell debris, cells were assessed for mean cell size based on forward scatter using flow cytometry. The mean forward scatter values were collected for each group (n = 4).

#### Transmission Electron Microscopy Analysis

Cells were cultured and stored in 24-well transwell polyethylene terephthalate membrane inserts (0.4 *µ*m pore size) (Corning, NY). Following storage, cell sheets were prepared for TEM imaging by fixing in 2.5% glutaraldehyde in 0.2 M cacodylate buffer adjusted to pH 7.4. They were post-fixed in 1% osmium tetroxide, and dehydrated through a graded series of ethanol up to 100%. The tissue blocks were immersed in propylene oxide twice for 20 minutes and embedded in Epon. Ultrathin sections were cut on a microtome (Leica Ultracut UCT; Leica, Wetzlar, Germany) and examined by transmission electron microscope (Model CM120; Philips, Amsterdam, The Netherlands). Micrographs were taken at a magnification of x24500.

### Phenotype

#### Proliferation, Apoptosis, Progenitor and Differentiation Markers Assessed by Immunocytochemistry

Cells were fixed, and prepared for immunocytochemistry staining following the protocol in Jackson *et al*.^17^. Antibodies and concentrations are listed in Table 2. Images were captured at random positions using an inverted epi-fluorescence microscope (Nikon Eclipse Ti with a DS-Qi1 camera; Nikon Instruments, Tokyo, Japan) at 200X magnification. The exposure length and gain were kept constant. ImageJ software was used to process the images. The percentage of positive staining for each marker was calculated based on an average from counting ~100 cells from randomly selected positions in n = 4 wells. Counts were verified by two independent investigators.

**Table 2:** Antibodies Used in Immunocytochemistry.

Antibody	Epitope	Supplier	Concentration
Proliferating Cell Nuclear Antigen (PCNA)	PC10 (mouse)	Dako	1:500
Cleaved Caspase-3	D175 (rabbit)	Cell Signaling	1:400
Tumor Protein P63 (P63)	EPR5701 (rabbit)	Abcam	1:300
Tumor Protein P63 (P63)	4A4 (mouse)	Abcam	1:100
ABC Transporter Family G2 (ABCG2)	BXP21 (mouse)	Santa Cruz	1:100
CCAAT/enhancer-binding protein delta (C/EBPδ)	Polyclonal (rabbit)	Abcam	1:600
Cytokeratin 14 (CK14)	LL002 (mouse)	Abcam	1:300
Cytokeratin 10 (CK10)	Polyclonal (rabbit)	Abcam	1:800
Involucrin	SY5 (mouse)	Santa Cruz	1:300

#### P63 Analysis by Cytospin

Cell suspensions from each group and control were fixed in ice-cold methanol at −20 °C for 7 minutes and loaded on to a glass slide by cytospinning at 800 g for 5 minutes. The slides were processed and analyzed using the immunocytochemistry protocol above for p63 [EPR5701] at 1:300 dilution.

#### Cytokine Profile Analysis by Multiple ELISA Tests

Medium was collected from non-stored control cells and each of the storage groups and frozen at −20C for later cytokine and growth factor analyses. ELISA analyses were carried out on samples and standards according to manufacturer instructions (Human MMP antibody array including all MMPs and TIMPs from Abcam, Cambridge, UK; IL-1α, IL-1β, IL-4 and IL-8 and EGF from Cloud-Clone Corp. Houston, USA; VEGF from Abbexa Ltd., Cambridge, UK; TGF-β1 and TGF-β3 from Assay Biotech, Sunnyvale, USA). Standard curves were made using the kit supplied standards and diluents. Background diluent controls were subtracted from readings. ELISA readings were taken using an Axon Gene Pix scanner at 450 nm.

#### Statistical Analysis

One-way ANOVA with Tukey’s post hoc pair-wise comparisons (SPSS ver. 19.0) was used to compare the groups. Data were expressed as mean ± SEM, and values were considered significant if *p* ≤ 0.05.

## Electronic supplementary material

Supplementary Table S1, Figures S1,S2 and S3
