# Supplementary material for: Optimization of Storage Temperature for Retention of Undifferentiated Cell Character of Cultured Human Epidermal Cell Sheets
Source: Sci Rep. 2017 Aug 15;7:8206. doi: 10.1038/s41598-017-08586-7 (PMC5557837; doi:10.1038/s41598-017-08586-7)
Supplement: Supplementary file 1 — Supplementary Table S1, Figures S1,S2 and S3 [file 41598_2017_8586_MOESM1_ESM.pdf]

Optimization of Storage Temperature for Retention of Undifferentiated Cell Character of Cultured Human Epidermal Cell Sheets.

Authors:
Catherine J. Jackson[1,2,3]*, Sjur Reppe[1], Jon R. Eidet[4], Lars Eide[1], Kim A. Tønseth[3,5], Linda H. Bergersen[2], Darlene A. Dartt[6], May Griffith[7], and Tor P. Utheim[1,2,3,4]
[1]Department of Medical Biochemistry, Oslo University Hospital, Oslo, Norway; [2]Institute of Oral Biology, Faculty of Dentistry, University of Oslo, Oslo, Norway; [3]Department of Plastic and Reconstructive Surgery, Oslo University Hospital, Oslo, Norway; [4]Department of Ophthalmology, Oslo University Hospital, Oslo, Norway; [5]Institute of Clinical Medicine, Faculty of Medicine, University of Oslo, Norway; [6]Schepens Eye Research Institute, Massachusetts Eye and Ear, Department of Ophthalmology, Harvard Medical School, Boston, USA; [7]Maissonneuve-Rosemont Hospital Research Centre and Dept. of Ophthalmology, University of Montreal, Montreal, Canada
*Corresponding author:
Catherine J. Jackson, Department of Medical Biochemistry, Oslo University Hospital, Kirkeveien 166, P.O. Box 4956 Nydalen, 0424 Oslo, Norway
Tel: +47 90224699; E-mail: catherinejoanjackson@gmail.com

Table S1

Group	% Viable Cells
Control	100
4°C	72
8°C	86
12°C	99
16°C	108
24°C	89

Table S1: Exact percentages of viable cells are presented to supplement Figure 2b

Figure S1

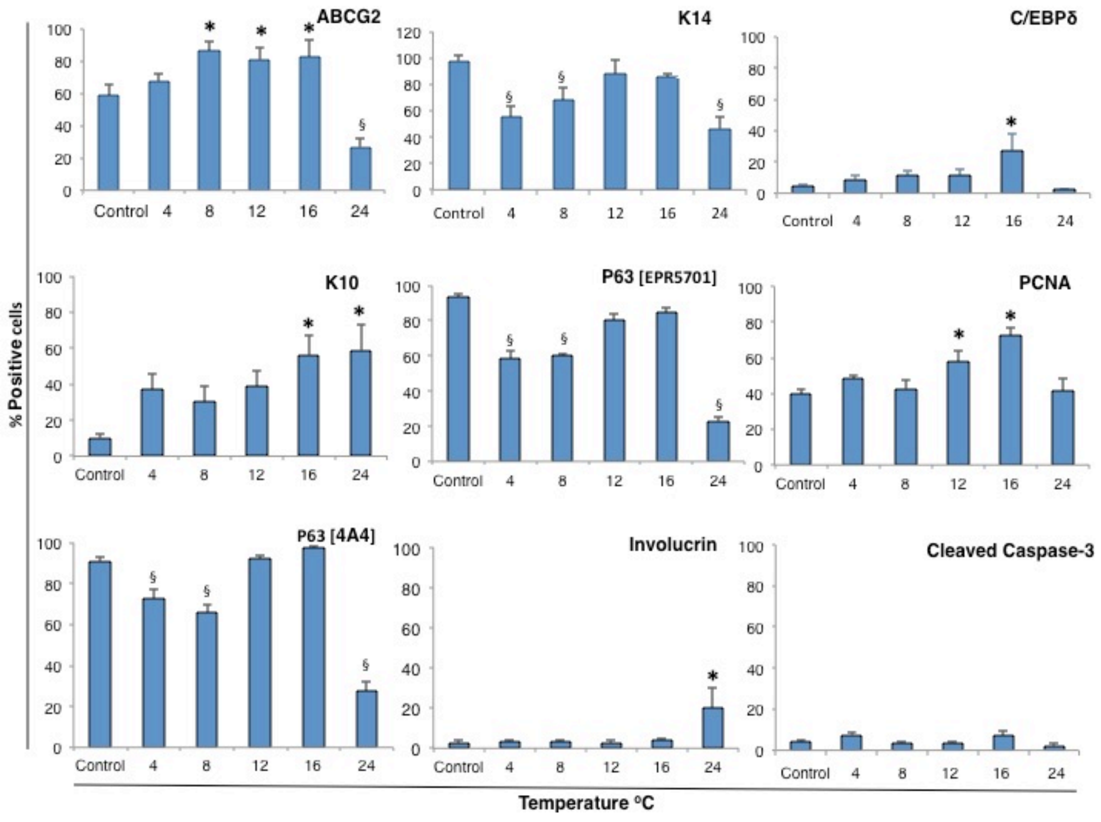

Figure S1: Marker expression in non-stored control and temperature groups. To supplement Figure 6 and Table 1, histograms show % positive cells for each marker (*= significantly increased compared to control; § = significantly decreased compared to control; $p \leq 0.05$).

Figure S2:
A)

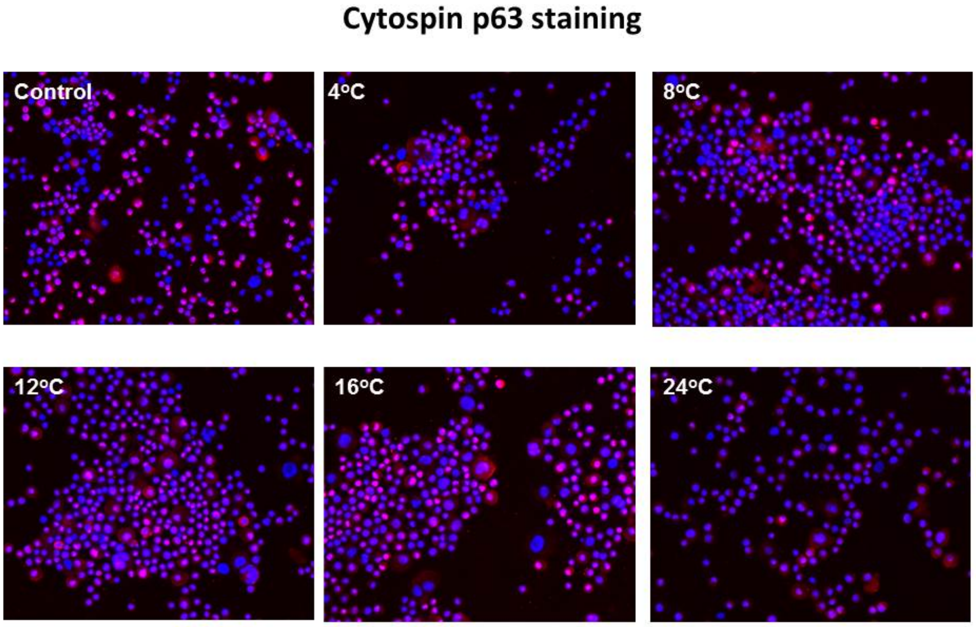

B)

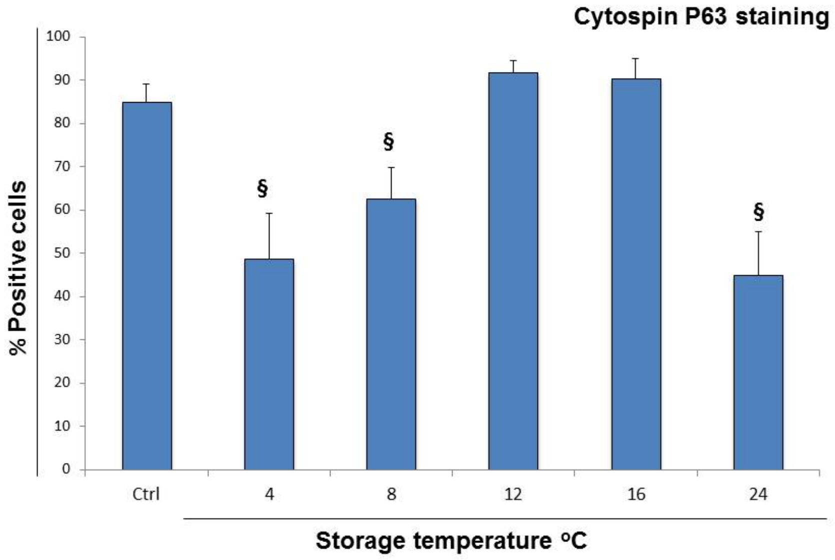

Figure S2: Cytospin staining of trypsinized cells from control and each temperature group.To supplement Figure 6 and Table 1, cells were trypsinized, fixed and placed onto a glass slide for immunocytochemistry analysis of P63 expression. This allowed visualization of dispersed cells from all layers. **A)** immunostaining with primary antibody **p63** and **Dapi. B)** histogram of P63 positive cells counted in four random areas (§ = significantly decreased compared to control (Ctrl); $p \leq 0.05$).

Figure S3

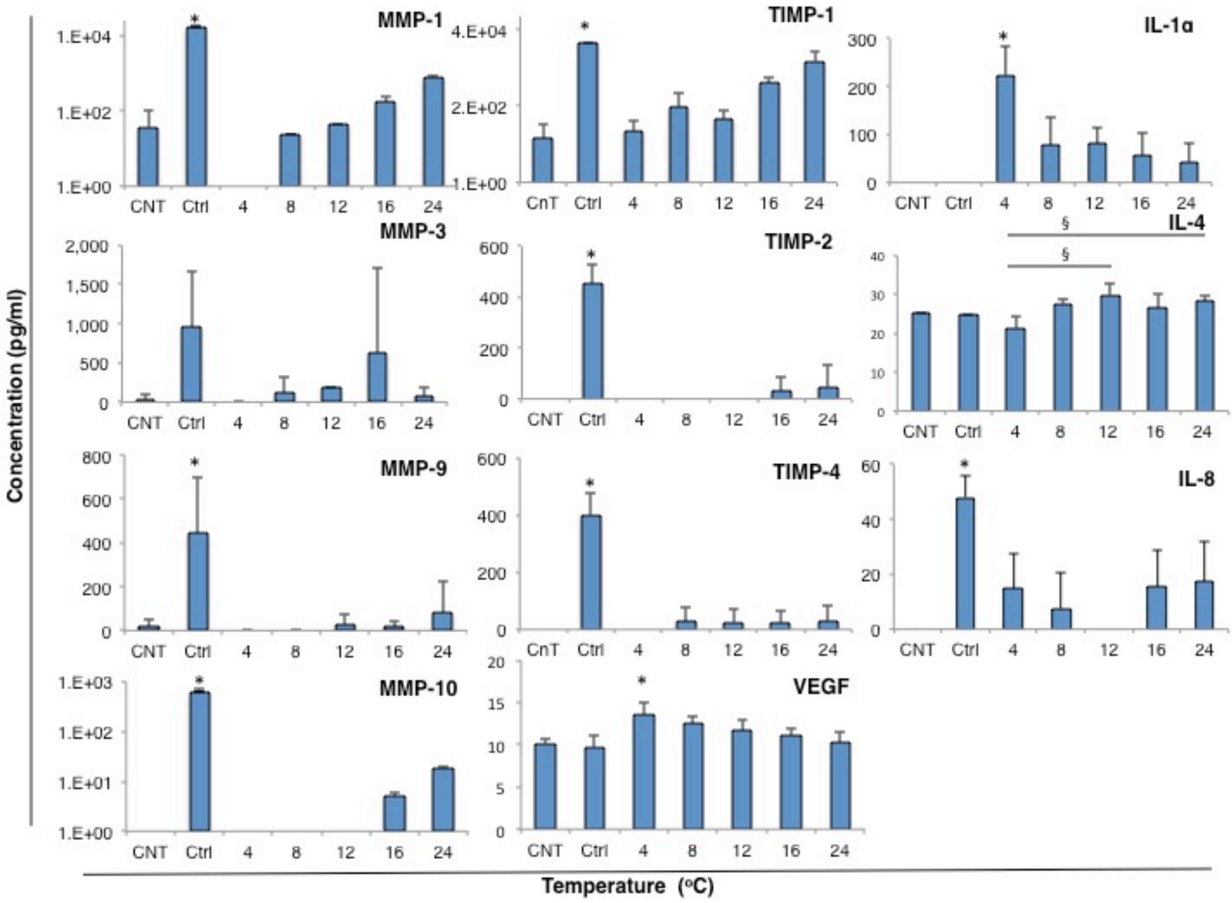

Figure S3: Cytokines and growth factors measured in culture medium (CNT-Prime) before storage and in storage medium following one-week storage. To supplement Figure 7, values for CNT-Prime and non-stored control media are shown separately. IL=interleukin; MMP=matrix metalloproteinase; TIMP=tissue inhibitor of matrix metalloproteinase; VEGF=vascular endothelial growth factor 165; CNT=CNT Prime culture medium (*= significantly increased compared to control (Ctrl); § = significantly decreased; $p \leq 0.05$).
